# Inter- and Intramolecular On-Surface Synthesis of Porphyrin-Based Nanostructures on Au(111) and Cu(111)

**DOI:** 10.1021/acsanm.5c01525

**Published:** 2025-06-10

**Authors:** Eleanor S. Frampton, Michael Clarke, Matthew Edmondson, Ailish Gray, Jonathan Bradford, Liv Warwick, Nicholas Pearce, Neil R. Champness, Alex Saywell

**Affiliations:** † MAX IV Laboratory, 226073 Lund University, 22100 Lund, Sweden; ‡ 6123 University of Nottingham, School of Physics and Astronomy, University Park, NG7 2RD Nottingham, U.K.; § The University of Birmingham, School of Chemistry, B15 2TT Birmingham, U.K.

**Keywords:** On-Surface Synthesis, Porphyrins, Photoelectron Spectroscopy (PES), Scanning Tunneling Microscopy (STM), Near-Edge X-ray Adsorption Fine Structure (NEXAFS), Temperature-Programmed X-ray Photoelectron Spectroscopy (TP-XPS)

## Abstract

Surface-confined synthesis provides alternative reaction pathways to those utilized within solution-phase chemistry and offers a route to extended molecular architectures with nanoscale dimensions and fascinating magnetic, electronic, and catalytic properties. However, these reaction pathways can be complex multistep processes, containing multiple reactive intermediates. Optimizing the selectivity and efficiency of such synthetic routes should be underpinned by detailed mechanistic insight, which requires submolecular spatial resolution in combination with details of chemical evolution throughout the reaction process. A key challenge is the application of an experimental methodology that allows in-depth study of multistep reactions. Here, we combine the spatial resolution of scanning tunneling microscopy with temperature-programmed photoelectron spectroscopies and present a comprehensive characterization of a multistep on-surface reaction utilizing a brominated porphyrin species. The porphyrin species employed is a highly functionalizable “molecular building block” from which nanostructured materials can be built, and within this work we identify key differences between the reaction on Cu(111) and Au(111). Intermolecular Ullmann-type coupling as well as intramolecular ring-closing and self-metalation are observed: specifically, on Au(111) we characterize self-metalation within covalently coupled assemblies of ring-closed TPP. Our results highlight the differing reactivity of Au(111) and Cu(111) and the strong influence of the substrate upon the reaction pathway and preferred products, and we provide spectroscopic and topographical characterization for all reaction steps.

## Graphical abstract

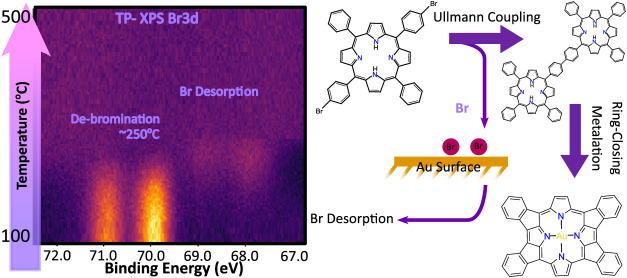

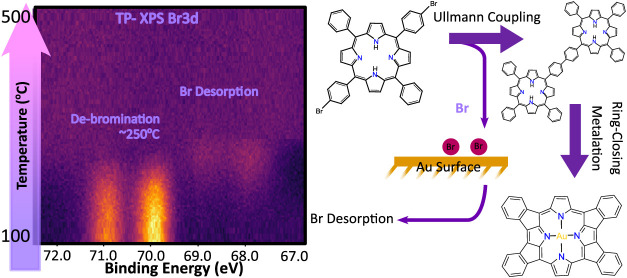

## Introduction

The synthesis of novel chemical species frequently progresses via complex reaction mechanisms. Such mechanisms may be considered as a sequence of elementary steps containing multiple transition states and reactive intermediates. The use of surface-confined reactions offers alternative reaction pathways to those typically available in solution phase chemistry and has been receiving much attention.^1–5^ The covalent nanostructures resulting from on-surface synthesis can display fascinating properties, such as π-magnetism in carbon-based structures,^6^ carbon capture in covalent frameworks,^7^ and the electronic properties of graphene nanoribbons.^8^ Indeed, on-surface synthesis has the potential to combine functional molecules to produce biomimetic systems for photocatalyis^9^ and bioinspired oxidation systems for organic synthesis.^10^ However, these novel reaction pathways are frequently multistep processes, containing multiple reactive intermediates with the possibility of side-products being generated (for example, consider the complexity of the electrochemical reduction of carbon dioxide^11^). Optimizing selectivity and efficiency of such synthetic routes is likely to be facilitated by detailed mechanistic insight, which requires submolecular spatial resolution combined with details of chemical evolution. Scanning probe microscopy techniques have successfully been employed to study the morphology changes during on-surface synthesis, down to the single-bond level,^3,12^ and when combined with photoelecton spectroscopy (PES) significant chemical, structural, and electronic insight may be obtained.^13–18^ Recently, there has been specific interest in “temperature-programmed” studies where PES techniques have been utilized to obtain kinetic information on on-surface processes.^19–21^

On-surface carbon–carbon bond formation for linking molecular precursors and creating bespoke nanostructures typically utilizes Ullmann-type coupling reactions,^13,22–25^ although other reaction pathways have been explored,^26,27^ and porphyrins are often employed as highly functional nanometer scale “molecular building blocks” ^2,15,28,29^ with their chemical properties being of significant interest.^30^ Studying on-surface reactions of porphyrins also introduces complexity as a myriad of inter- and intramolecular processes may occur. For example, the intramolecular process of electrocyclic ring closing has been reported for phenyl substituted porphyrins.^31–35^ Similarly, the process of self- or *trans*-metalation is frequently observed for free base and metalated porphyrins deposited on metal surfaces.^36–39^

Here we study the on-surface reactions of a halogenated tetraphenyl porphyrin, Br_*x*_TPP (*x* = 0–4), on Au(111) and Cu(111). A combination of scanning tunneling microscopy (STM) and PES techniques are employed which provide insight into the structural changes at various stages in the reaction and the chemical evolution of the system (characterized by temperature-programmed X-ray photoelectron spectroscopy, TP-XPS). We provide a comparative study of the reaction processes on these two different substrates and highlight the role of the substrate with respect to multistep on-surface synthesis involving functionalized porphyrin species. The chemistry of the two surfaces is known to drive intermolecular reactions, including covalent coupling on Au(111) and the formation of metal–organic structures on Cu(111),^1,4,5^ and we show here how a sequence of inter- and intramolecular reactions progress on each substrate. We discuss key differences in terms of reaction steps: debromination, formation of extended (covalent or metal–organic) structures, ring-closing, and self-metalation. Specifically, we observe a sequence of reaction steps on Au(111) resulting in the formation of covalently coupled chains of TPP that have undergone covalent coupling, ring-closing, and metalation.

## Results and Discussion

### Structural Evolution: STM Characterization

Br_*x*_TPP was initially studied via low-temperature (4.7 K) STM on Au(111) and Cu(111) substrates. Submonolayer coverages were prepared via thermal sublimation under ultrahigh vacuum (UHV) conditions (Au(111) and Cu(111) surfaces held at 30 °C and between −170 and −130 °C, respectively, during deposition; see the Experimental Section for full details). Samples underwent sequential annealing stages, with STM characterization of the resultant molecular structures detailing the progression of the Ullmann-type on-surface reaction following each heating stage (see Figure 1a for an overview of the proposed reaction pathway). The progression of the reaction from the as-deposited precursor species via (i) debromination, (ii) formation of extended structures (metal–organic frameworks (MOFs) and covalent organic frameworks (COFs)), (iii) ring-closing, and (iv) self-metalation are characterized and discussed in detail below.

**Figure 1:**
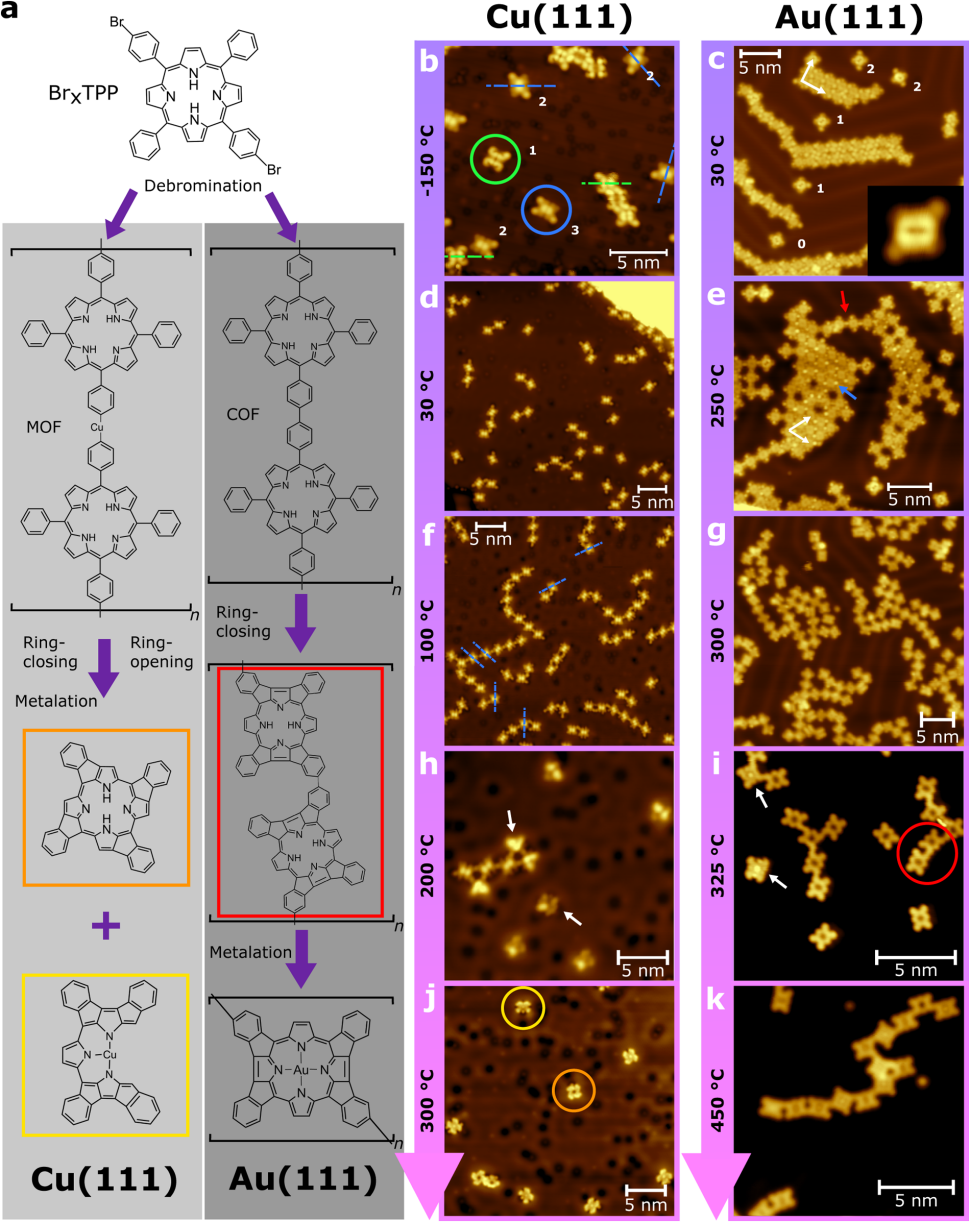
Summary of the on-surface reaction of Br_*x*_TPP on Cu(111) and Au(111). (a) The proposed reaction pathways for *trans*-Br_2_TPP on Cu(111) and Au(111) with accompanying STM topographs (b–k) for each reaction step; see text for description. STM image parameters are detailed in Table S1.

Characterization of Br_*x*_TPP on both Au(111) and Cu(111) following deposition reveals species with 0, 1, 2, 3, and 4 attached bromine atoms (*x* = 0–4) (examples indicated in Figure 1b,c) with the position of each Br being clearly identified as a bright protrusion in agreement with extensive STM studies.^28,29,40^ Thermal decomposition of the C–Br bond has previously been reported for evaporation temperatures of 315 °C or higher,^28^ but is not expected under the conditions utilized here (250 °C). *trans-*Br_2_TPP (from herein simply termed Br_2_TPP) is most frequently observed on the surface following deposition (Figure S1); within solution-phase synthesis Br_2_TPP is the expected majority product^41,42^ and it is unsurprising that this is most frequently observed within the STM measurements.

On Au(111) Br_*x*_TPP is observed as isolated individual molecules and close-packed structures with lateral dimensions extending to tens of nanometers (Figure 1c). Individual molecules are pinned at “elbow” sites^43^ on the herringbone reconstruction, while close-packed structures, with square unit cells (θ = 90 ± 5°), reside within the face-centered cubic (*fcc*) regions of the surface reconstruction.^44^ The stabilization of nonhalogenated TPP species has previously been assigned to π–H interactions.^14^ Here the presence of halogen atoms may provide additional stabilization via a halogen-bonding motif^45,46^ (Figure S2). Individual molecules display a contrast generally assigned to a “saddle shaped” conformation (see inset in Figure 1c),^40,47,48^ where two of the four pyrrolic rings are tilted toward the surface and two are tilted away due to conformational flexibility imbued by the rotations possible around multiple bonds within the porphyrin core.

Deposition of Br_*x*_TPP on Cu(111) (cooled to approximately −150 °C) results in significant structural differences as compared with adsorption upon Au(111). Extended islands are not observed, with the majority of porphyrins found as isolated species or small clusters (as previously observed^49^). Molecules are preferentially oriented such that symmetry planes of the molecule are found at 60° rotations relative to each other (see dashed blue lines in Figure 1b and Figure S3) assigned to an alignment with the ⟨110⟩ close-packed atomic row directions on the (111) surface (in agreement with previous combined STM and DFT studies;^40^ see Figure S4). The absence of extended self-assembled structures is assigned to a strong molecule–substrate interaction and dipole formation.^49^ Interestingly, molecules are observed with two distinct appearances (indicated by green and blue circles in Figure 1b and Figure S3). These two different structures have been attributed to the presence of two distinct conformations, “saddle” (as discussed above) and “inverted”:^50–53^ where the iminic pyrrole rings display a near 90° rotation out of the plane of the molecule, breaking the aromaticity of the macrocycle, with the iminic nitrogen atoms within pyrrolic rings pointing directly toward the surface, facilitating a strong interaction with the substrate. Here, the inverted structure is the most frequently observed conformer, with a relative abundance of 75% (*N* = 233), although the exact proportions are likely preparation dependent as a transition from “saddle” to “inverted” has been observed for elevated sample temperatures (about −100 °C^53^).

#### Debromination and Intermolecular Reactions

Following annealing (to 250 and 30 °C, for the Au(111) and Cu(111) surfaces, respectively), the debromination step of the Ullmann-coupling reaction is initiated. On Au(111) debromination and covalent coupling results in the expected formation of polymer chains^28,29^ (Figure 1e, red arrow indicates covalently bound porphyrins). Bright features within the two-dimensional close-packed domains, indicated by the blue arrow, are assigned to Br atoms still attached to the pendent phenyl rings (i.e., complete dehalogenation has not occurred at this stage). The close-packed islands exhibit a rhomboidal unit cell (θ = 60 ± 5°; indicated in Figure 1e and Figure S5). Increased annealing temperature (300 °C) results in further debromination on Au(111), loss of the close-packed islands, and formation of branched polymers due to the mixture of degrees of bromination (and structural isomers) within the starting material (Figure 1g). The separation between the porphyrin units within the polymer is measured to be 1.7 ± 0.1 nm, in excellent agreement with prior experimental work and DFT calculations for the covalently bonded structure.^28^

On Cu­(111), annealing to 30 °C gives rise to debrominated monomers and short oligomers (e.g., dimers and trimers), which we assign to the formation of C–Cu–C metal–organic interactions (Figure 1d; supported by XPS analysis showing that complete debromination is expected following annealing to 30 °C *vide infra*). This process is in agreement with the wealth of literature reporting on-surface Ullmann coupling where MOF structures containing adatoms are formed.^4,13^ Heating in a stepwise manner to higher temperatures (∼100 °C) results in longer MOFs (Figure 1f) with features visible between neighboring porphyrins (assigned to Cu adatoms;^54^ see Figure S6). Within the MOFs, individual molecules retain the same polar orientation as for the as-deposited species (60° rotations, blue dashed lines in Figure 1f). Interestingly, all molecules now display an identical contrast, assigned to the “inverted” adsorption geometry,^50,52,53^ within the 1D MOFs. As expected all reaction steps occur at a lower temperature on Cu(111) as compared to Au(111).^55^

#### Intramolecular Reactions (Ring-Closing and Metalation)

Following the formation of quasi-1D structures (MOFs or COFs), additional heating results in intramolecular reactions of the tetraphenyl porphyrin species. On Au(111), ring-closing reactions, where new C–C bond formation occurs between phenyl groups and the porphyrin macrocycle, are observed after annealing to 325 °C (Figure 1i, red circle indicates proposed ring-closed structure indicated by red box within [Fig fig1]a), as previously reported for isolated porphyrins.^14,31,36^ Here we find that ring-closing occurs within covalently bonded polymer chains, demonstrating the sequential nature of the on-surface reaction and the relative stability of the C–C coupling between neighboring porphyrins compared to the barrier for cyclodehydrogenation which gives rise to the ring-closed porphyrinoids. In addition, the occurrence of limited metalation is indicated by the appearance of bright features at the center of the porphyrin macrocycle (indicated by white arrows in Figure 1i), and further heating (∼450 °C) results in almost complete metalation of porphyrins within the covalently coupled chains (Figure 1k).

On Cu(111) annealing from ∼30 to ∼300 °C facilitates three processes: (i) heating to ∼200 °C results in the breakup of MOF structures (Figure 1h), (ii) individual molecules now exhibit a distorted appearance (bright features assigned to incomplete ring closing,^14,31^ as indicated with an arrow within Figure 1h), and (iii) heating to ∼250 °C or higher facilitates the formation of a variety of ring-closed, ring-opened,^32^ and metalated structures (Figures 1j and S7). The “pinwheel” structure (highlighted by an orange circle) and the ring-opened metalated structure (yellow circle) are assigned based on related studies,^32^ with a total of four main products observed (see Figure S7). However, the ring-opened-metalated species (yellow circle) is the most common, accounting for 54% (*N* = 105) of molecules (structure proposed in ref 32). Metalation is suggested by the appearance of a bright protrusion within the macrocycle and is supported by the XPS analysis presented below.

### Temperature Dependent Studies: XPS Characterization

STM topographs provide detailed spatial characterization of structural changes during on-surface reactions. However, low-temperature STM topographic studies provide a static “snapshot” of topography within a reaction and are not ideally suited to monitor chemical evolution (although “videos” of sequential STM topographs may capture on-surface processes^44,56^ ). XPS can provide details of chemical changes during a reaction,^15,19–21^ and here we provide details of distinct reaction steps by utilizing a combination of TP-XPS and STM.

#### Debromination

The initial debromination step can be resolved within TP-XPS: a series of XP spectra are acquired while heating the surface at a set rate (Table S2) and combined to produce a “heat-map” where the *x*- and *y*-axes represent binding energy and substrate temperature, respectively, and the color contrast represents measured photoelectron intensity (see Figure 2a). Characterization of the Br 3d XPS region over the temperature range −180 to 300 °C shows that on Cu(111) the as-deposited Br_*x*_TPP exhibits dominant features at 70.35 and 71.38 eV binding energy (BE) [NB two peaks are expected for a single chemical environment due to spin–orbit splitting (3d_3/2_, 3d_5/2_)]. The intensity of these peaks abruptly decreases at −25 °C alongside the simultaneous appearance of peaks at 68.68 and 69.71 eV BE. In agreement with previous studies of C–Br bond cleavage on metallic substrates,^57,58^ the pair of peaks at higher BE are assigned to the intact C–Br environment, with the lower BE pair assigned to Br at the surface (Cu–Br). This bromine then remains on the surface for the duration of the reaction. The slight shift to lower BE for the C–Br species over the temperature range −160 to −25 °C is tentatively assigned to an interaction with Cu adatoms prior to cleavage of the C–Br bond. Individual XP spectra before/after cleavage of the C–Br bond are shown below/above the heat map within Figure 2a (see also Figure S8 and Table S4). Low intensity peaks (68.68 and 69.71 eV BE) are visible for the as-deposited sample and are assigned to Cu–Br species formed by deposition of bromine from the evaporation source or by cleavage of the C–Br bond at more reactive surface features (e.g., low coordinated atomic sites at step-edges where the reaction can happen at reduced thermal energy compared with (111) terraces^59^).

**Figure 2:**
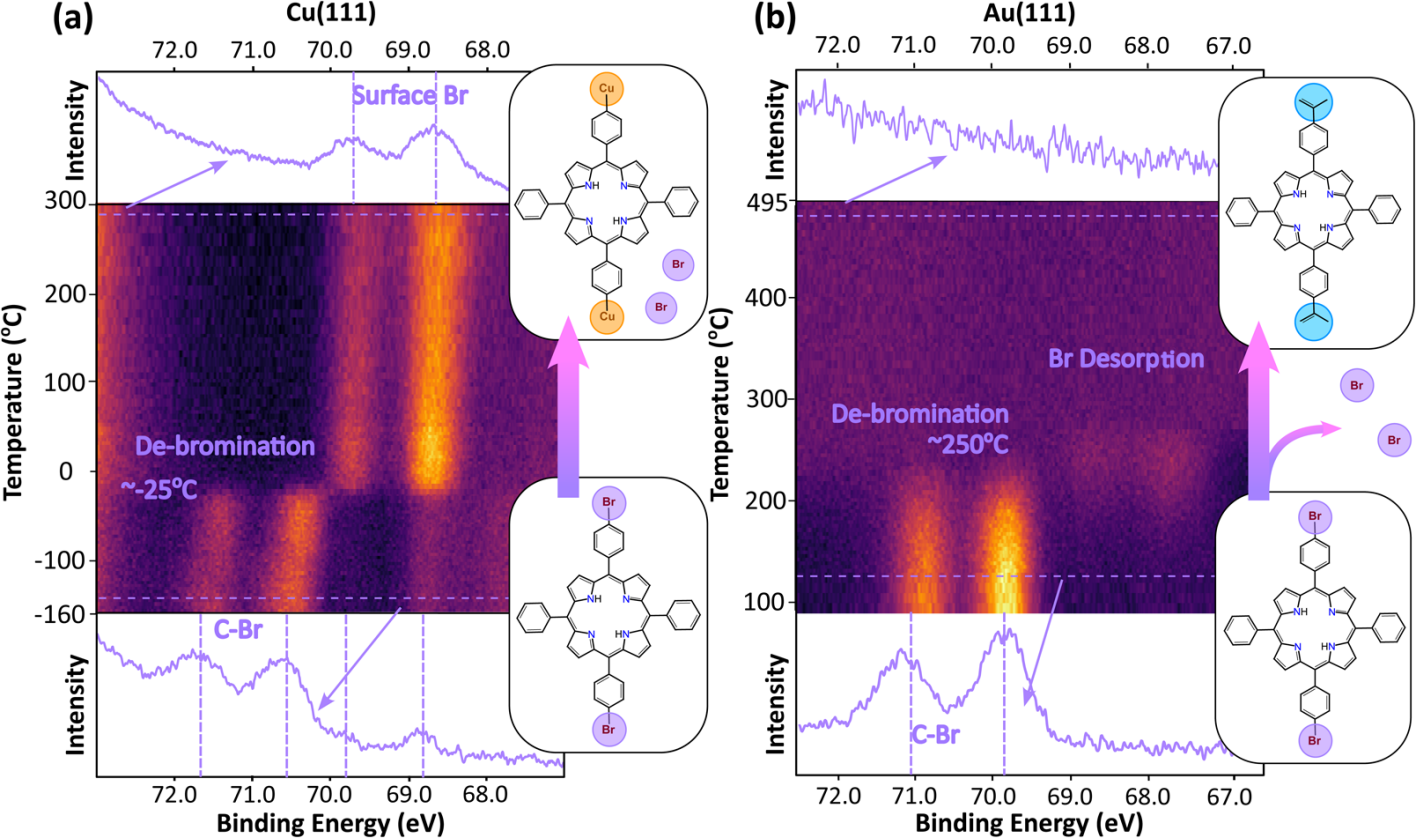
Temperature-programmed XPS (TP-XPS) measurements of the Br 3d region. (a) Br_*x*_TPP on Cu(111) over the temperature range −180 to 300 °C and (b) Br_*x*_TPP on Au(111) over the temperature range 100 to 500 °C. Line profiles show the initial and final states. Color contrast shows intensity of photoelectron signal (arbitrary units). All spectra measured with photon energy = 380 eV and pass energy = 50 eV within fixed mode.

TP-XPS data for the same reaction on Au(111) (Figure 2b) shows that the high BE peaks (C–Br), BEs 69.82 and 70.86 eV, are present following deposition (substrate temperature 25 °C) indicating intact Br_*x*_TPP.^60^ The C–Br peaks for the as-deposited molecules display a slightly lower binding energy on Au(111) compared with the same species on Cu(111). The C–Br features decrease in intensity and vanish upon heating to ∼250 °C; similar to the reaction on Cu(111), this is assigned to complete debromination of Br_*x*_TPP. The peaks which emerge at lower binding energy (67.78 and 68.81 eV BE) with weak intensity are assigned to a transient Au–Br species. This transient Au–Br species vanishes as the temperature rises to 300 °C and is assigned to the desorption of a bromine containing species (likely to be HBr,^61–63^ where intramolecular ring-closing may provide a source of hydrogen for the formation of HBr). This transient species, not observed in STM, indicates that a Au–Br species is formed following the C–Br bond cleavage.

#### Metalation

The interaction between the nitrogen atoms within the core of the porphyrin macrocycle and the substrate can be studied via TP-XPS by focusing on the N 1s core level. Intact Br_*x*_TPP possesses two nitrogen environments, iminic (−N=) and aminic (−N–H), which appear as two distinct peaks in the XP spectra. Figure 3a (lower spectrum, see also Figure S9 and Table S5) shows the N 1s region for intact Br_*x*_TPP on Cu(111) with peaks at BEs of 397.93 eV (iminic) and 399.8 eV (aminic) in agreement with prior studies.^15,16^ On Au(111), the appearance of the initial state is similar to that on copper, with iminic and aminic peaks observed at 397.1 and 399.2 eV, respectively (Figure 3b, lower spectrum). Secondary peaks at 397.8 eV (iminic) and 399.9 eV (aminic) arise from an interaction between the TPP macrocycles and Au adatoms.^14,47^ The separation between the BE of the iminic and aminic peaks is larger on Au(111) than Cu(111), likely due to a stronger interaction between the Cu surface and the iminic nitrogen.^50^

**Figure 3:**
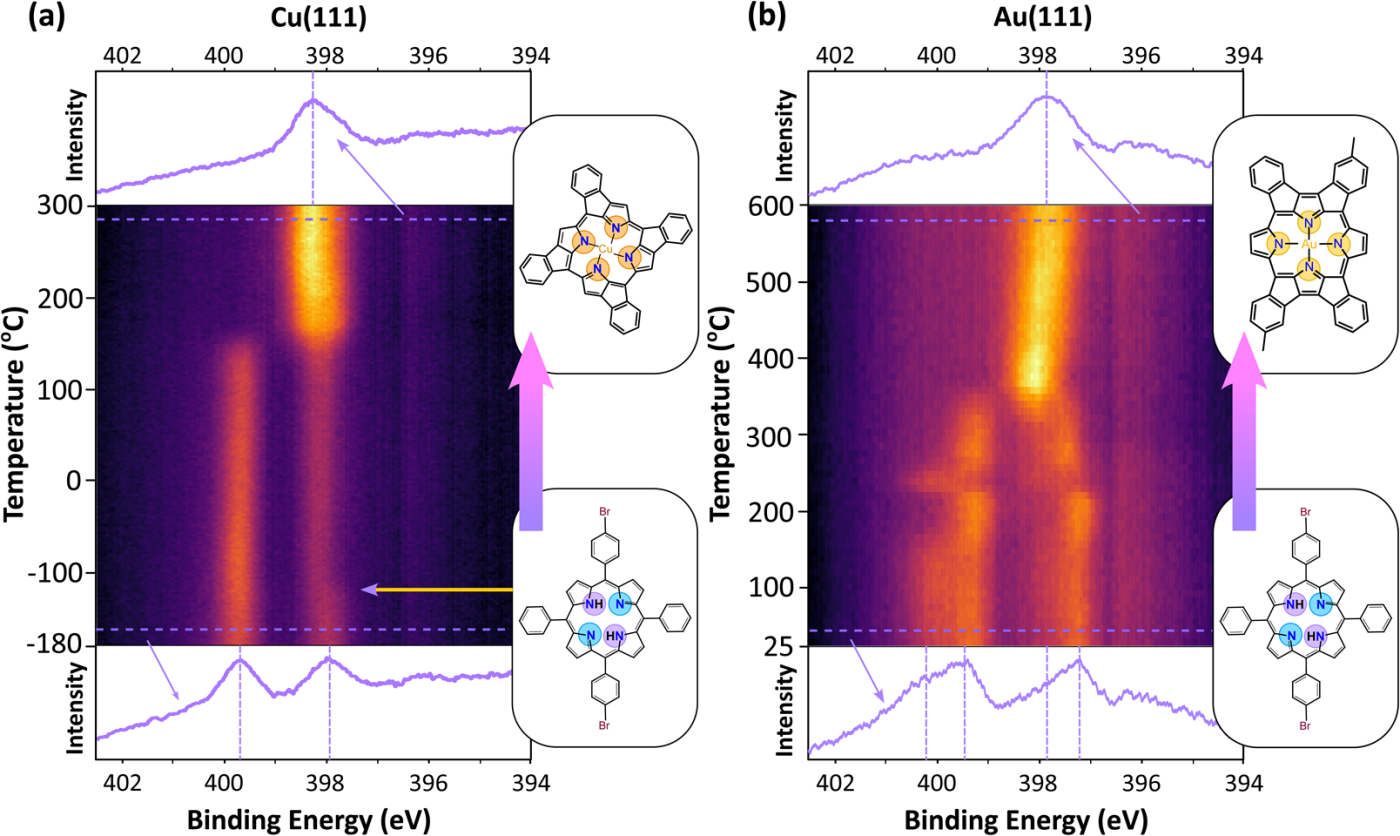
Temperature-programmed XPS measurements of the N 1s core level for (a) Br_*x*_TPP on Cu(111) over the temperature range −180 to 300 °C and (b) Br_*x*_TPP on Au(111) over the temperature range 25 to 500 °C. Spectra show the initial and final states. Color contrast shows intensity of photoelectron signal (arbitrary units). All spectra measured with photon energy = 500 eV and Ep = 50 eV in fixed mode.

The N 1s TP-XPS map showing the evolution of Br_*x*_TPP on Cu(111) is shown in Figure 3a, where two distinct processes are observed to occur. The first change occurs at approximately −100 °C, where the position in the peak at 397.93 eV BE (assigned to the iminic nitrogen^52^) shifts 0.15 eV toward higher binding energy (indicated by the yellow arrow in Figure 3a). Such a shift may be attributed to Br_*x*_TPP molecules transforming from the saddle conformation to the inverted conformation,^50–52^ with the iminic nitrogen atoms pointing directly toward the surface; our prior study has demonstrated that such a transition may be observed and characterized using STM.^53^ The strong interaction with the surface, resulting in the inverted conformation, is likely to give rise to some of the observed differences in the reaction pathways on Cu(111) and Au(111), as seen in Figure 1. The inverted conformation may be less sterically compatible with the covalent coupling of porphyrins which would account for the preference of MOF formation on Cu(111).

A second step is observed to occur at around 150 °C where the aminic peak has disappeared entirely, leaving a broad feature with a peak at 397.26 eV BE which is assigned to a metalated porphyrin (Cu atom bonded to all four nitrogen atoms). Such self-metalation of porphyrins on metals is a well-known phenomenon.^14,16,36–38,64,65^

On Au(111), the presence of four nitrogen environments (see lower spectrum in Figure 3b) introduces additional complexity to the metalation, but the progress is similar to that observed on Cu(111). The TP-XPS map for the N 1s region on Au(111), Figure 3b, shows conversion to Au-TPP at temperatures over 400 °C. In addition, the shoulders to the high BE side of the aminic and iminic nitrogen peaks (corresponding to the interaction with a Au adatom) display discontinuities during the temperature ramp. We observe sequentially the loss of the Au-adatom peak, the shift to higher BE for the imininc (−N=) peak, and the growth of a peak at 398.0 eV assigned to a metalated Au-TPP structure.

### Chemical Changes within the Carbon Structure

The changes to the bromine and nitrogen chemical environments, captured by TP-XPS and detailed above, provide information about the progression of the on-surface reactions of Br_*x*_TPP on Au(111) and Cu(111). Significant changes also occur within the carbon backbone of the molecule, as visible within STM characterization. Here we provide a spectroscopic signature of these reaction steps by considering the C 1s region of the XPS.

The C 1s spectra for as-deposited Br_*x*_TPP, on both Cu(111) and Au(111), exhibit a broad asymmetric shape arising from the multitude of carbon environments (Figure 4a,d). Six distinct chemical environments are assigned based upon the wealth of porphyrin studies reported upon in the literature (assignments are indicated within the chemical structures in Figure 4; see Table S3 for peak fitting details). The peaks to the high BE side of the carbon envelope, 285.9 eV on Cu(111) and 285.8 eV on Au(111), diminish upon heating and are assigned to an intact C–Br bond^22,58,60^ (pink peaks labeled CBr in Figure 4a–f). In line with the thermally induced cleavage of the Br–C bonds observed in the Br 3d TP-XPS (Figure 2), we see a reduction in the C–Br features (pink) at high BE within the C 1s spectra on both the Cu(111) and Au(111) surfaces following annealing (Figure 4b,e). The appearance of a distinct peak at low BE (283.39 eV, green peaks labeled CCu) on the Cu(111) surface is assigned to the presence of an organometallic C–Cu species (supporting the assignment of a MOF within the STM analysis; Figure 1f).^13,66,67^ On Cu(111), further annealing (300 °C, Figure 4c) results in the loss of the C–Cu peak (in agreement with the break up of MOF structures observed within the STM analysis). In addition to the assigned chemical environments, we ascribed a broad feature at the high BE side of the main peak (286–287 eV) to a shakeup (S-u) feature.

**Figure 4:**
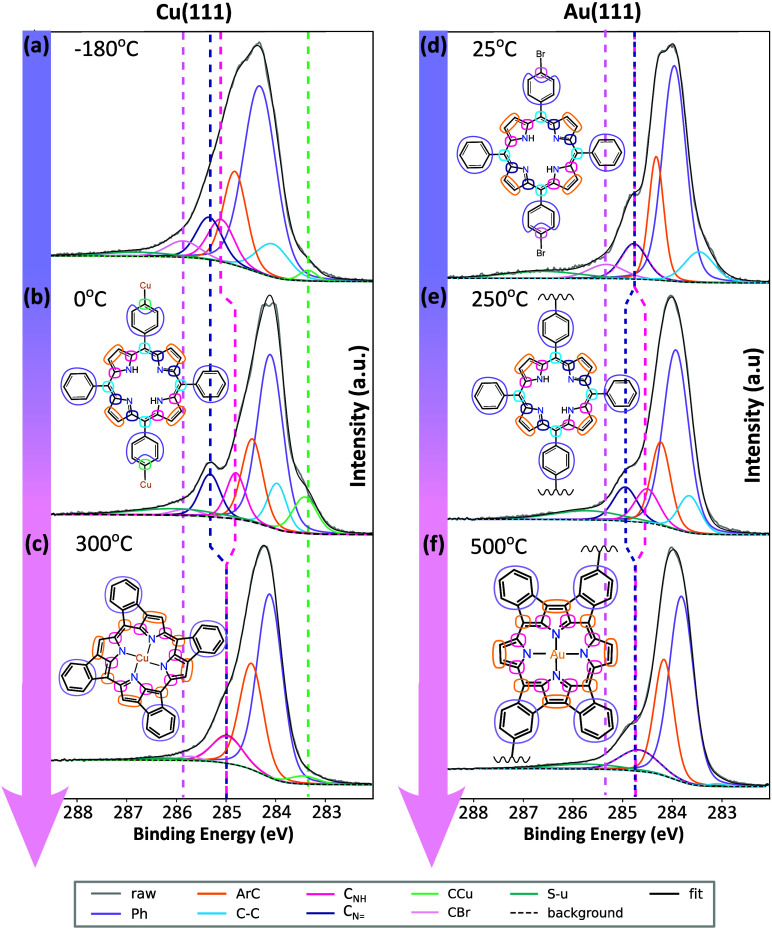
High resolution XPS of the C 1s core level and peak fitting for three stages within the on-surface reaction on Cu(111) [(a) as-deposited and following annealing to (b) 0 and (c) 300 °C] and Au(111) [(d) as-deposited and following annealing to (e) 250 and (f) 500 °C]. The assigned carbon environments are indicated on the inset chemical structures (see legend)). C 1s XP spectra were measured at photon energy = 380 eV and Ep = 50 eV.

Information on the chemical state of the nitrogen species can also be gained from a characterization of the neighboring carbon atoms: carbon atoms adjacent to the −N= and −N–H nitrogen environments are expected to exhibit different BEs when the chemical environment of the nitrogen species varies (e.g., strong interaction with substrate atoms).^17^ On Cu(111), where a strong interaction between −N= and the substrate has previously been observed,^50–52^ peaks at 285.3 and 285.1 eV BE (blue and red peaks in Figure 4a) are assigned to carbon species adjacent to −N= and −N–H (labeled here as *C*_*N*_ and *C*_*NH*_). On Au(111), where a strong interaction between −N= and substrate is not expected, a single peak at 284.8 eV is assigned (red and blue peaks in Figure 4d). This distinction between *C*_*N*_ and *C*_*NH*_ is an important feature and provides details of the progress of the on-surface reaction following annealing. On Cu(111), annealing to 0 °C induces a widening of the splitting between the two species (0.51 eV separation, with peaks at 285.29 and 284.78 eV, Figure 4b); this agrees with the expected strong interaction between the iminic nitrogen and the substrate within the inverted structure.^50,51^ Further annealing to 300 °C (Figure 4c) results in the coalescence of the *C*_*N*_ and *C*_*NH*_ environments, which is in agreement with the appearance of a metalated (Cu-TPP) species as observed in the N 1s TP-XPS and STM data. Further annealing, resulting in cyclization (ring-closing) and metalation reactions on both the Cu(111) and Au(111), is aligned with the loss of the C–C feature (Figure 4c,f) and the merger of the *C*_*N*_ and *C*_*NH*_ environments into a single peak.

### NEXAFS

Near-edge X-ray absorption fine structure (NEXAFS) spectra provide information on electronic structure (for unoccupied states), and angularly resolved data provides insight into structural/conformational changes that occur during the reaction.^68^ Angle dependent NEXAFS measurements at the C K-edge were performed at various stages of the reaction on both Cu(111) and Au(111). Figures 5a,b show the C K-edge NEXAFS spectra for Br_*x*_TPP on both Au(111) and Cu(111), respectively, in their initial (as deposited) and final states of the reaction (following ring-closing and metalation). For porphyrin species, the C K-edge measurements probe unoccupied π* states associated with the macrocyclic core, providing details on the average orientation of the core relative to the surface (Figure 5c shows the fitting of the angle resolved data peak positions and intensities provided in Table S6).

**Figure 5:**
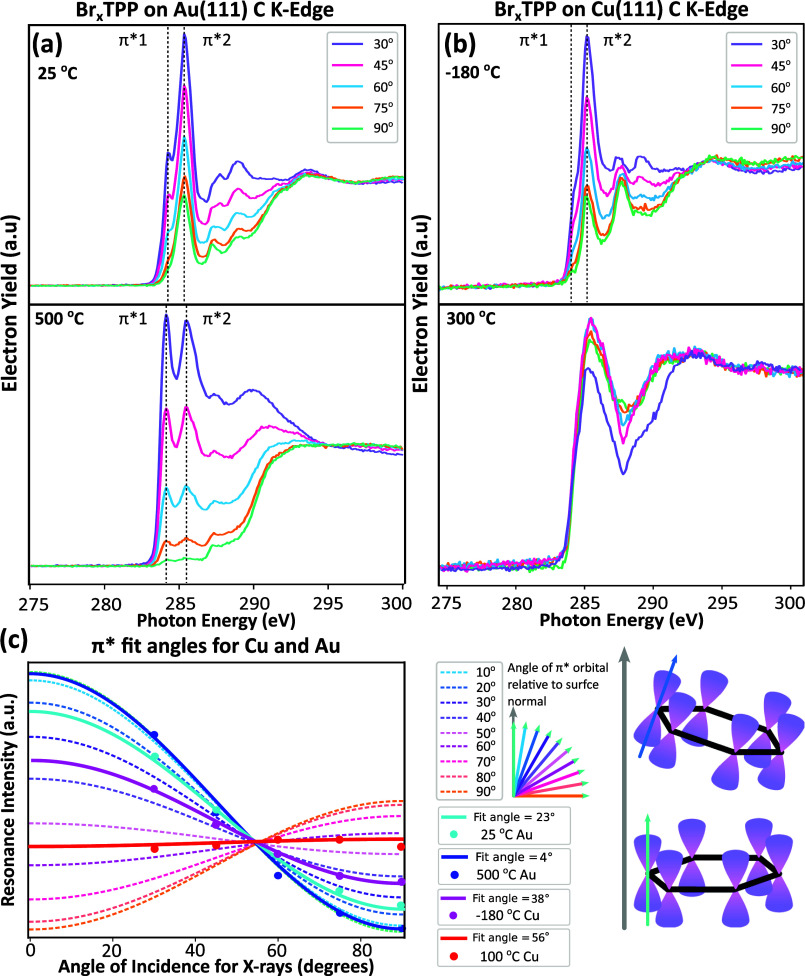
NEXAFS spectra at the carbon K-edge showing initial and final states of the reaction on (a) Au(111) and (b) Cu(111); measured with incident light at 30°, 45°, 60°, 75°, and 90°. Spectra were measured in PEY with a retardation potential of 220 V. (c) Fitting of the angle resolved data of the π* resonance to extract the average angles of the carbon skeleton relative to the surface.

In the as-deposited state on Au(111) (Figure 5a, top), several distinct peaks are present (see Table S6). Upon annealing to the final state (500 °C, Figure 5a, bottom), the spectra show several changes. By considering the first resonance (labeled as π*1) and the associated dichroism we can obtain the orientation of the molecular plane relative to the substrate (see Figure S10 and Table S7); Figure 5c shows the angular dependence of the intensity of the π*1 resonance for as-deposited (cyan, 25 °C sample temperature), 23° tilt relative to surface plane, and following anneal (blue, anneal to 500 °C), 4° tilt relative to the surface plane. The average tilt angle measured for the initial state (23°) is consistent with a porphyrin molecule adopting the saddle conformation (commonly observed on Au(111)).^69^ In the final state the tilt angle of 4° is consistent with the ring-closing reaction discussed above, which results in planarization, carbon–carbon bonds forming between the pendent phenyl rings and the core, and a nominal 0° tilt angle.

On Cu(111) NEXAFS spectra for the as-deposited Br_*x*_TPP display several distinct peaks (Figure 5b; see Table S6 for peak assignment) which are significantly altered upon annealing to 300 °C (STM data indicates molecules are now in a ring-closed/-open and metalated state). The angular variation in the intensity of the π*2 resonance (Figure 5c) yields average tilt angles of 38° for the as-deposited species (magenta, −180 °C sample temperature) and 58° following annealing (red, anneal to 100 °C). For the initial state, a mixture of saddle and inverted conformations^50,51^ may result in the higher average tilt angle, as compared to on Au(111) where only the saddle conformation is expected to be present. In the final state, the tilt angle of 58° is likely to be reflective of a range of molecular orientations. This is consistent with the panoply of ring-closed, ring-opened, and metalated species observed within STM analysis following annealing. We speculate that the strong N–Cu interaction and associated inverted structure underlie the range of structures formed on Cu(111). On Au(111) where this interaction is weaker, the formation of planar ring-closed species is likely to be more facile.

## Conclusion

In this study we demonstrate the effects of the choice of substrate on the progression of on-surface reactions of Br_*x*_TPP. The combination of scanning probe microscopy and X-ray spectroscopy facilitates an in-depth characterization of a multistep reaction process at each stage. This approach allows the structural and chemical changes of molecular species (as isolated units and within extended nanostructures) to be addressed as “snap-shots” of the reaction via STM and “real time” chemical changes taking place to be observed by employing TP-XPS measurements. Additionally, by utilizing angular dependent NEXAFS measurements, we were able to observe conformational changes within the molecule during the reaction process.

We have identified key differences between the reaction progression and formation of nanostructures on Cu(111) and Au(111) and have highlighted how intermolecular Ullmann-type coupling, intramolecular ring-closing, and self-metalation progress on these substrates (providing spectroscopic fingerprints for the observed reaction steps). The nature of the interaction between the substrate and the nitrogen atoms within the porphyrin macrocycle drives specific conformational structures, with the inverted structure favored on Cu(111) and the saddle structure observed on Au­(111). Differences in reaction pathway on the two substrates are likely to be influenced by steric properties of these two conformations; for example, the saddle shaped conformation on Au(111) appears to facilitate intramolecular ring closing within covalently bonded polymers when compared to Cu(111). The self-metalation for covalently coupled assemblies of ring-closed TPP on Au(111), which is absent on Cu(111), may also be a direct consequence of the prevalence of saddle-shaped confirmation. The ability to characterize multiple, and often competing, on-surface processes and their reactive intermediates (including details of their chemical state and conformation) is essential in the future development of efficient reaction pathways for functional nanomaterials.

## Experimental Section

### Synthesis

The porphyrin, Br_*x*_TPP, was prepared using literature methods.^42^

### STM Measurements

Scanning tunneling microscopy (STM) imaging was performed using a Scienta Omicron POLAR low-temperature STM system, operating under ultrahigh vacuum (UHV) conditions. The system has a base pressure of sub 3 × 10^–10^ mbar. The STM was cooled with liquid helium (4.7 K). All STM images were measured in constant current mode with electrochemically etched tungsten tips that may be coated in gold or copper during tip optimization. This was achieved via controlled indentation into the Au(111) and Cu(111) substrates. The Au(111) and Cu(111) single crystal surfaces were prepared by cycles of Ar ion sputtering for 30 min at 1.0 keV, followed by annealing at 500 °C for 30 min. Annealing temperatures were estimated by thermocouple measurements close to the heating stage. The molecules were deposited (using a Kentax UHV Evaporator heated to 250 °C) onto the cleaned surfaces. The Au(111) surface was held at room temperature, and the Cu(111) was held at reduced temperature during molecular deposition.

### Spectroscopy

Au­(111) and Cu(111) surfaces were both prepared under UHV conditions, base pressure 7.7 × 10^–10^ mbar, by cycles of sputtering and annealing (sputter 1 keV for 20 min, anneal to 550 °C, and held for 5 min before returning to room temperature). The Cu(111) surface was annealed in 2 × 10^–6^ mbar of O_2_. The resultant clean surfaces were characterized with XPS to show no contaminant species present.

The Br_*x*_TPP molecules were heated in a Knudsen-type evaporator held at 250 °C, and the surfaces were then exposed to the flux for 15 min to achieve submonolayer coverage. The Au(111) surface was held at room temperature during the deposition while the Cu(111) surface was held at −180 °C during the deposition.

All spectroscopy experiments were performed at MAX IV in Lund, Sweden, at the Surface and Materials Science (SMS) Branch of the FlexPES beamline.^70^ FlexPES is situated on the 1 GeV storage ring and uses a planar undulator and collimated plane-grating monochromator to deliver photons with energy range 40–1500 eV. The beam spot on the SMS branch was defocused to produce a 1 × 0.4 mm^2^ spot. The defocused beam was used for all measurements reported to limit radiation damage to the samples. No radiation damage was detected on either of the samples. Base pressure did not exceed 1 × 10^–9^ mbar in the experimental chamber during the experiment.

The experimental station at FlexPES features a Scienta SES-2002 hemispherical analyzer (Scienta Omicron) positioned 40° from the incident photon beam, which was used for the photoelectron spectroscopy. PES measurements were taken at normal emission. A home-built partial electron yield (PEY) microchannel plate (MCP) detector was used for the X-ray absorption measurements. NEXAFS measurements were taken in both total electron yield (TEY) via sample drain current and also with the partial electron yield (PEY) MCP detector. The PEY measurements are presented here. For the N K-edge, a retardation potential of 300 V was used, and for the C K-edge a potential of 220 V was used. On Au(111), all measurements aside from the TP-XPS were carried out at RT, while on Cu(111), all measurements aside from the TP-XPS were carried out at −180 °C.

## Supplementary Material



## Data Availability

Data for this article, including STM, XPS, and NEXAFS data files, are available at The University of Nottingham Research Data Management Repository at 10.17639/nott.7538.

## Notes

Additional experimental details, including STM characterization, XPS and TP-XPS characterization, and NEXAFS characterization (PDF)
