# Supplementary material for: Inter- and Intramolecular On-Surface Synthesis of Porphyrin-Based Nanostructures on Au(111) and Cu(111)
Source: ACS Appl Nano Mater. 2025 Jun 10;8(24):12608–18. doi: 10.1021/acsanm.5c01525 (PMC12186228; doi:10.1021/acsanm.5c01525)
Supplement: Supplementary file 1 [file an5c01525_si_001.pdf]

Inter- and Intramolecular On-Surface Synthesis of Porphyrin-Based Nanostructures on Au(111) and Cu(111)

Eleanor S. Frampton[1,], Michael Clarke[2], Matthew Edmondson[2], Ailish Gray[2], Jonathan Bradford[2], Liv Warwick[2], Nicholas Pearce[3], Neil R. Champness[3] and Alex Saywell[2*]*

[1]Max IV Laboratory, Lund University, 22100 Lund, Sweden

[2]School of Physics & Astronomy, The University of Nottingham, Nottingham, NG7 2RD, UK.

[3]School of Chemistry, The University of Birmingham, Birmingham, B15 2TT, UK

*Corresponding authors: EF (Eleanor.Frampton@maxiv.lu.se) & AS (Alex.Saywell@nottingham.ac.uk)

Supporting Information

Contents

Details of STM characterisation: Br_xTPP monomers and self-assembled structures 2
Details of XPS characterisation: TP-XPS and peak assignment 6
Details of NEXAFS characterisation: Peak assignment and discussion of N K-edge spectra 10

Details of STM characterisation: Br_xTPP monomers and self-assembled structures

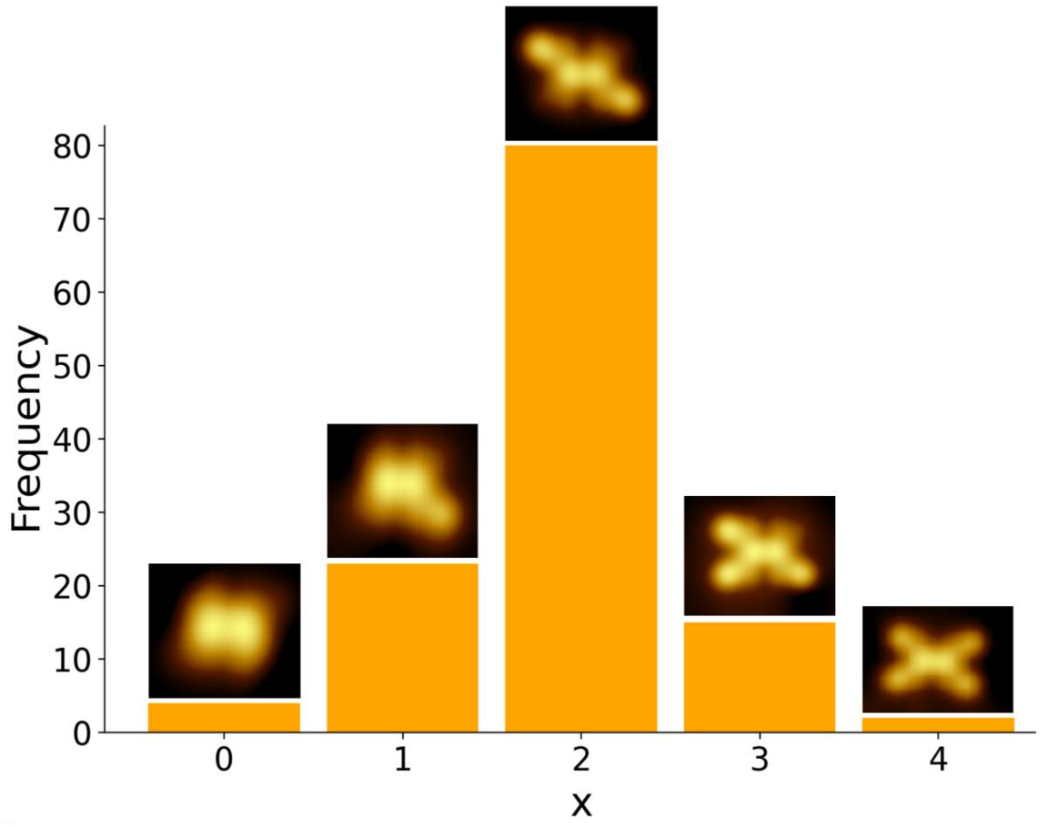

Figure S1: Histogram showing the distribution of the number of bromines, *x*, per deposited Br_xTPP molecule observed on the Cu(111) surface. Images are representative STM topographs for each species *x* = 0-4. Sample size of N = 124. STM parameters $V_{sample-bias}$ = -1.60 V, $I_{set-point}$ = 403 pA (*x*=0-3) and 500pA for *x* = 4.

The use of Br_xTPP allows the effect of structural isomers, and degree of functionalisation (number of Br atoms as functional groups), upon the progression of the reaction to be studied. In agreement with previous work,[1] we observe that the structure of the covalently coupled network is driven by the symmetry of the Br_xTPP species (e.g. BrTPP forms dimers, Br_4TPP forms a square planar node), but no preferential link is observed between the conformation ('saddle' or 'inverted') and the degree of functionalisation or nature of structural isomerisation.

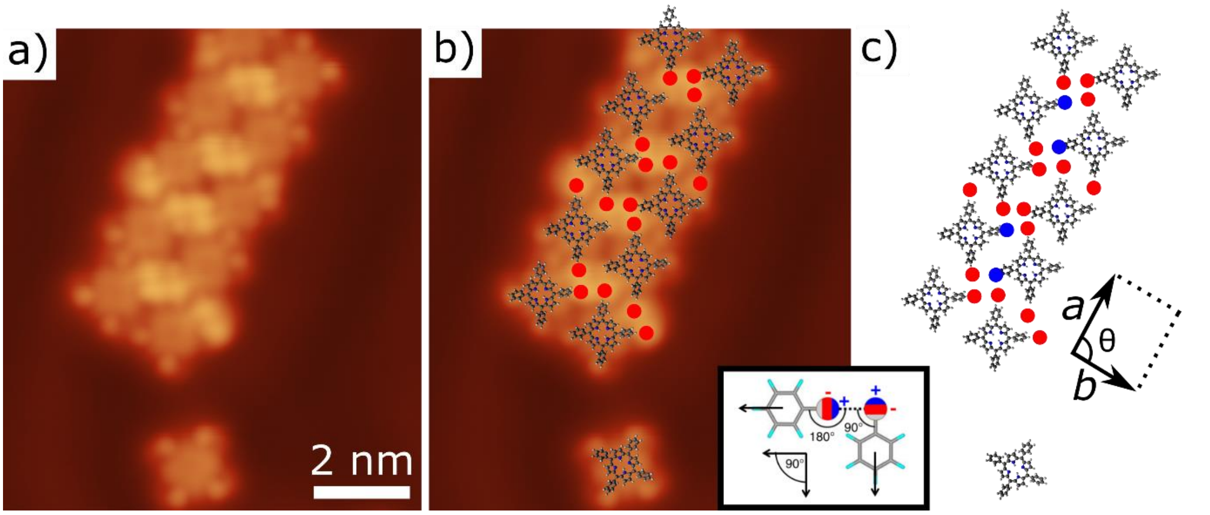

Figure S2: Details of close-packed Br_xTPP on Au(111). (a) Shows a close-up of two close-packed islands of BrxTPP within the *fcc* (face centered cubic) regions of the surface. (b) The molecular overlayer with indicated and scaled BrxTPP are displayed on top of the island from (a) with bromine atoms indicated as red dots. (c) shows the same overlayer structure as (b) with potential position of bromine atoms indicated as blue dots. Inset shows the interaction of halogen atoms on Au(111), indicating a 90° bonding angle that may drive molecular self-assembly (taken from Tschakert *et al.* [2] CC BY 4.0). Image parameters: (a-b) $V_{sample\text{-}bias}$ = 0.3 V and $I_{set\text{-}point}$ = 200 pA.

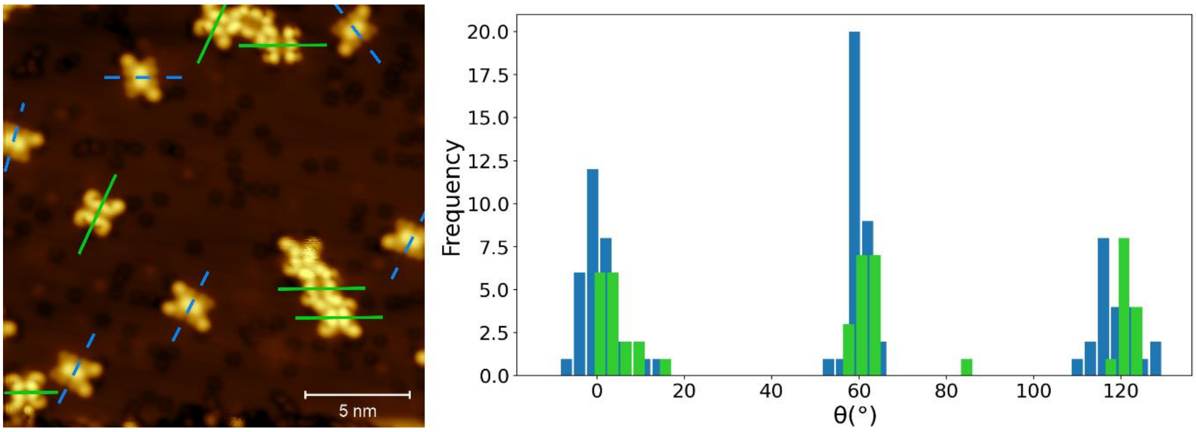

Figure S3: Details of the alignment of Br_xTPP on Cu(111). (Left) STM topograph of the two conformations of Br_xTPP ('saddle' and 'inverted') on Cu(111). The dashed blue lines and solid green lines show the relative orientations of the molecules. (Right) Histogram showing the angular distribution of the alignments of the two conformers (green = 'saddle', blue = 'inverted'). Molecules are observed at 60° rotations to one another indicating a potential preferential alignment with the <110> close-packed atomic row directions on the (111) surface. STM parameters $V_{sample\text{-}bias}$ = -1.60 V, $I_{set\text{-}point}$ = 403 pA.

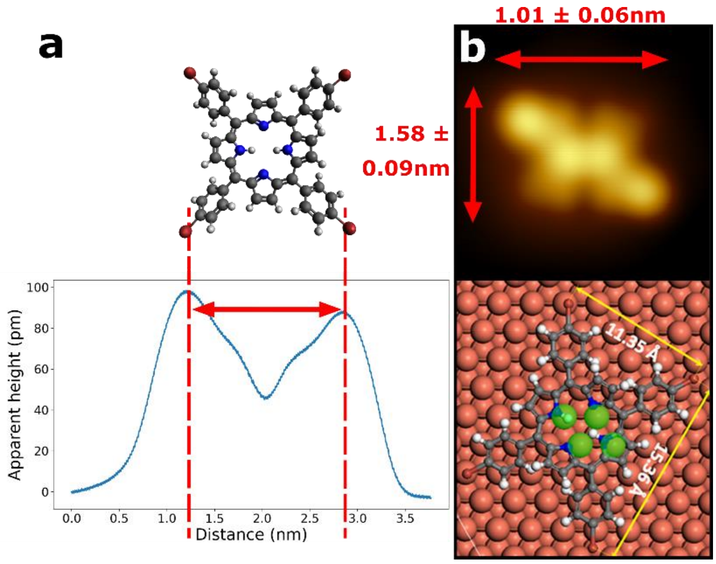

Figure S4: Dimensions and alignment of Br_xTPP on Cu(111). a) Line profile of the separation between two bromine atoms on Br_xTPP. b) Comparison between the experimentally measured line profiles and a DFT calculated structural model of the inverted conformation (*N*=16 for horizontal dimension and *N*=22 for vertical dimension) [lower part of (b) reproduced from [3] CC BY 4.0]. STM parameters $V_{sample\text{-}bias}$ = -1.60 V, $I_{set\text{-}point}$ = 200 pA.

Table S1: Values of the STM imaging parameters (sample bias, *V*, and current set-point, *I*) for the STM topographs presented within Figure 1 of the main manuscript.

Figure 1 – sub-panel	Sample Bias (V)	Current set-point (pA)
b	-1.60	200
c	0.50	100
d	-1.60	342
e	-0.50	300
f	-1.60	200
g	0.50	300
h	-1.60	179
i	0.50	100
j	-1.60	338
k	-1.40	100

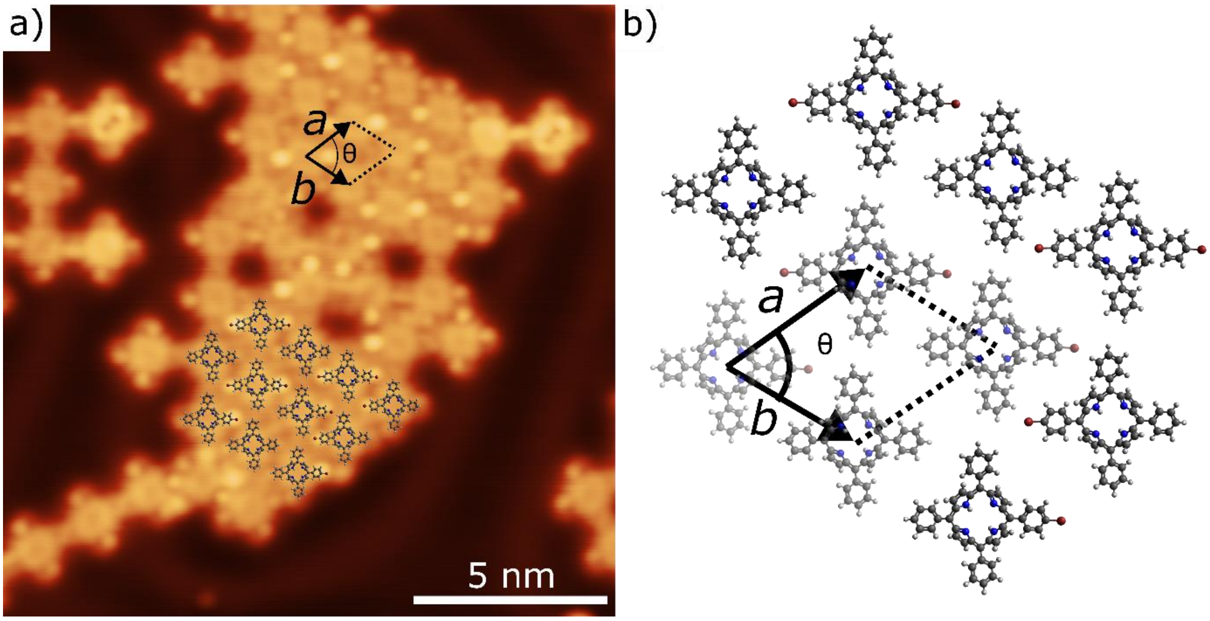

Figure S5: Rhomboidal close-packed assembly of Br_xTPP on Au(111) following annealing to 250°C. a) STM topograph showing a close-packed island of Br_xTPP formed within the *fcc* regions of the surface. b) Proposed structure, and measured dimensions, for the molecular arrangement in (a): $\theta = 60 \pm 5°$, $a = 1.5 \pm 0.10$ nm, b = 1.3 0.1 nm.

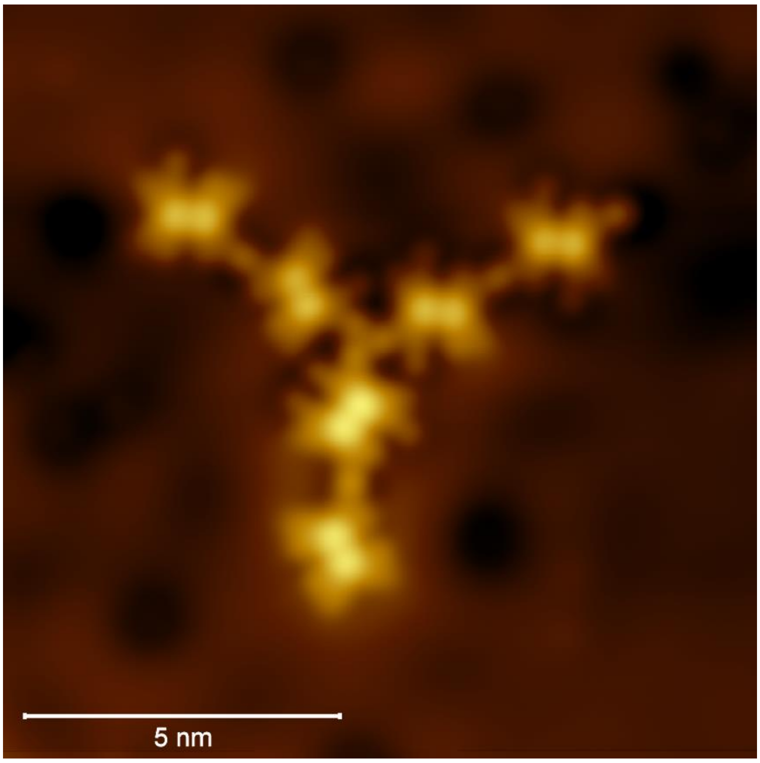

Figure S6: STM topograph showing the formation of a metal-organic framework (MOF) formed by Br_xTPP on Cu(111) following annealing at 150°C. The features between neighboring porphyrins are assigned to metal adatoms. STM parameters: $V_{sample\text{-}bias} = -1.60$ V, $I_{set\text{-}point} = 304$ pA.

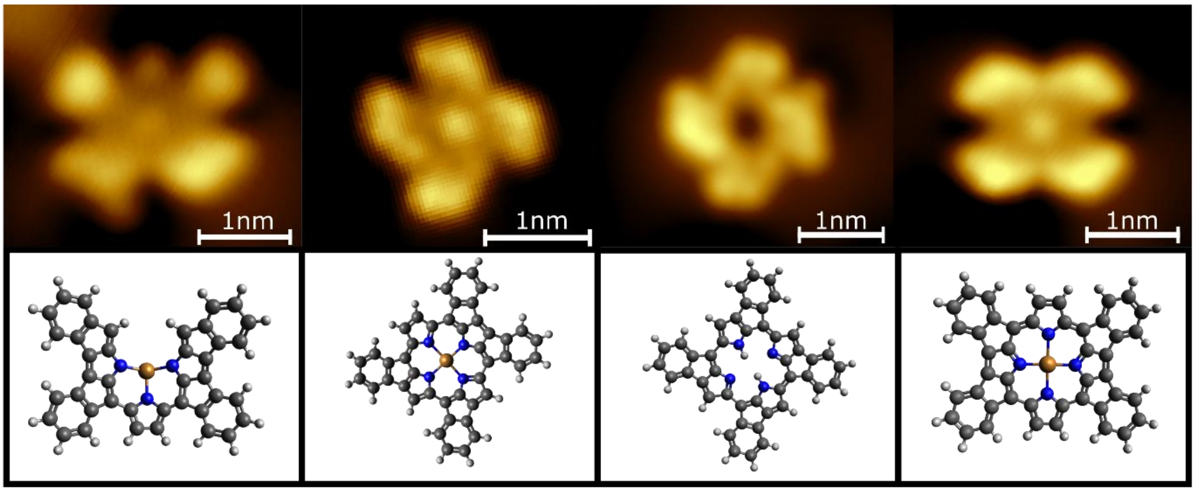

Figure S7: Details of the four main ring-closing and metalated structures formed from Br_xTPP on Cu(111) following annealing to 250°C. STM topographs are presented above proposed structural models (based upon references discussed within the main manuscript). STM parameters: $V_{sample\text{-}bias}$ = -1.6 V, with $I_{set\text{-}point}$ = 211, 338, 294, 294 pA (from left to right).

Details of XPS characterisation: TP-XPS and peak assignment

Table S2: Details of TP-XPS presented within figures 2 and 3 within the main manuscript.

Experiment	Substrate	Ramp Rate	Regions	Temp Range (°C)	No. of spectra
1	Cu(111)	5 K/min	C1s & Br	-160 to 200	97
2	Au(111)	5 K/min	C1s & Br	25 to 500	85
3	Cu(111)	2 K/min	N1s	-180 to 300	174
4	Au(111)	5 K/min	N1s	25 600	6

Table S3: Details of C1s XPS assignment – list BEs, no. of atoms, assignments, and include figures larger versions of the chemical structures in Figure 4 of the manuscript.

**Cu(111) at -180 °C**								
**Name**	Ph	C-C	ArC	CN	CNH	CBr	CCu	S-u
**Position**	284.3	284.1	284.8	285.3	285.1	285.9	283.3	286.8
**FWHM**	0.8	0.8	0.6	0.6	0.6	0.8	0.4	1.6
**Atoms**	20-22	4	8	4	4	2-4	0-1	
**Cu(111) at 0 °C**								
**Name**	Ph	C-C	ArC	CN	CNH	CBr	CCu	S-u
**Position**	284.1	283.9	284.4	285.3	284.8	285.8	283.4	286.0
**FWHM**	0.6	0.5	0.5	0.5	0.5	0.7	0.6	1.9
**Atoms**	18-20	4	8	4	4	0	4-6	
**Cu(111) at 300 °C**								
**Name**	Ph	C-C	ArC	CN	CNH	CBr	CCu	S-u
**Position**	284.1		284.5	285.0	285.0			286.3
**FWHM**	0.6		0.6	0.7	0.7			1.4
**Atoms**	24		12	4	4			

**Au(111) at 25 °C**								
**Name**	Ph	C-C	ArC	CN	CNH	CBr	CAu	S-u
**Position**	284.0	283.5	284.3	284.8	284.8	285.3		286.6
**FWHM**	0.6	0.7	0.4	0.6	0.6	0.9		1.8
**Atoms**	20-22	4	8	4	4	2-4		
**Au(111) at 250 °C**								
**Name**	Ph	C-C	ArC	CN	CNH	CBr	CAu	S-u
**Position**	284.0	283.7	284.3	284.9	284.6			285.8
**FWHM**	0.6	0.5	0.5	0.6	0.6			1.5
**Atoms**	24	4	8	4	4			
**Au(111) at 500 °C**								
**Name**	Ph	C-C	ArC	CN	CNH	CBr	CAu	S-u
**Position**	283.8		284.2	284.7	284.7			286.0
**FWHM**	0.6		0.5	0.9	0.9			1.5
**Atoms**	24		12	4	4			

We estimate throughout this article that our binding energy values are correct to within 20 meV, however in the C1s region we have quoted our peak fitting binding energies with an accuracy of within 100 meV due to the overlapping peaks in the fit.

To account for the variation in x the relative intensity of the CBr peak was allowed to vary such that it could correspond to between 0 and 4 CBr species per molecule. Acceptable fits were achieved with values corresponding to between 2 and 4 CBr species per molecules (in agreement with the STM analysis which indicates that the x=2 species occurs in the highest proportion – see Fig. S1.)

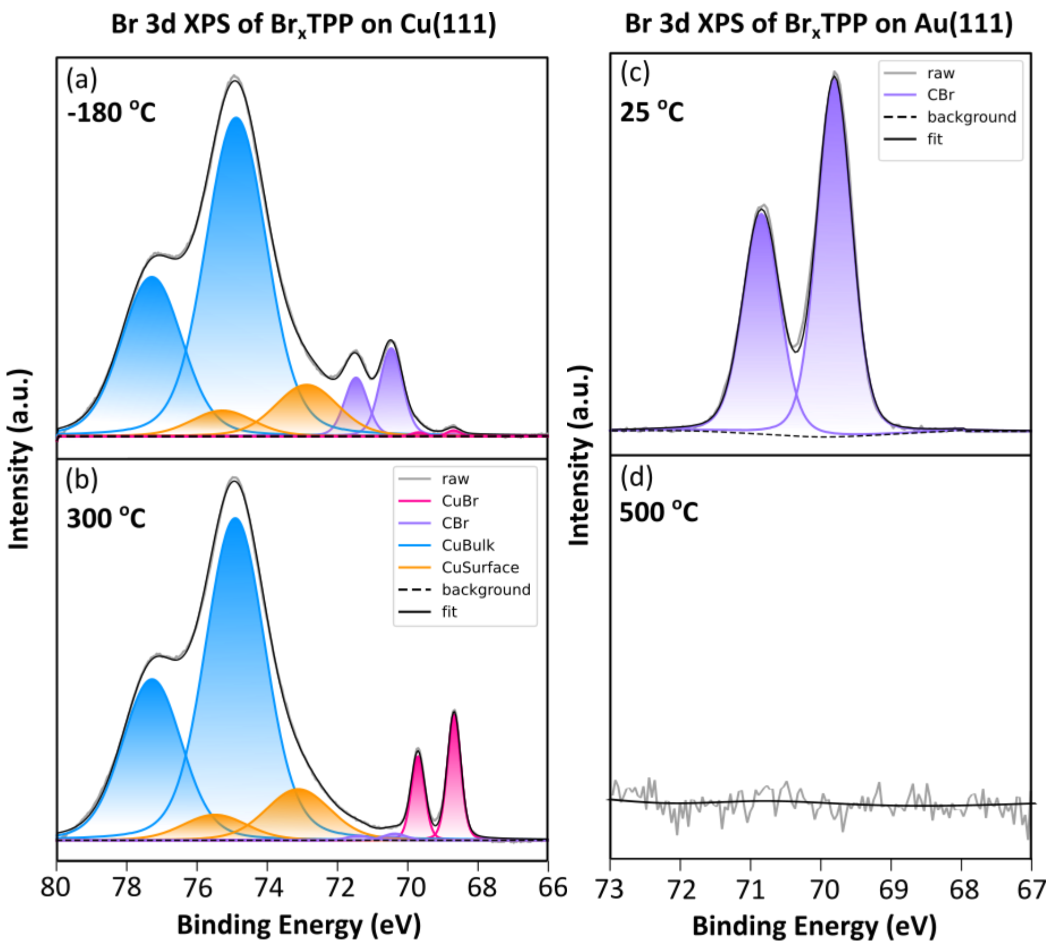

Figure S8: Br 3*d* XPS region. High resolution XPS measurements and peak fitting for initial and final states of reaction. (a,b) Br_xTPP on Cu(111) at -180 °C and 300 °C, Br3d core level, photon energy= 170 eV, (c,d) Br_xTPP on Au(111) at 25 °C and 500 °C Br3d core level, photon energy= 170 eV.

Table S4: BE of peaks assigned to Br 3d region in Figure S8

	**Br-C**			**Br-M (M=Au,Cu)**		
	**BE (eV)**		**FWHM**	**BE (eV)**		**FWHM**
**Cu(111)**	68.68	69.71	0.36	70.45	71.48	0.76
**Au(111)**	69.83	69.97	0.61	-	-	-

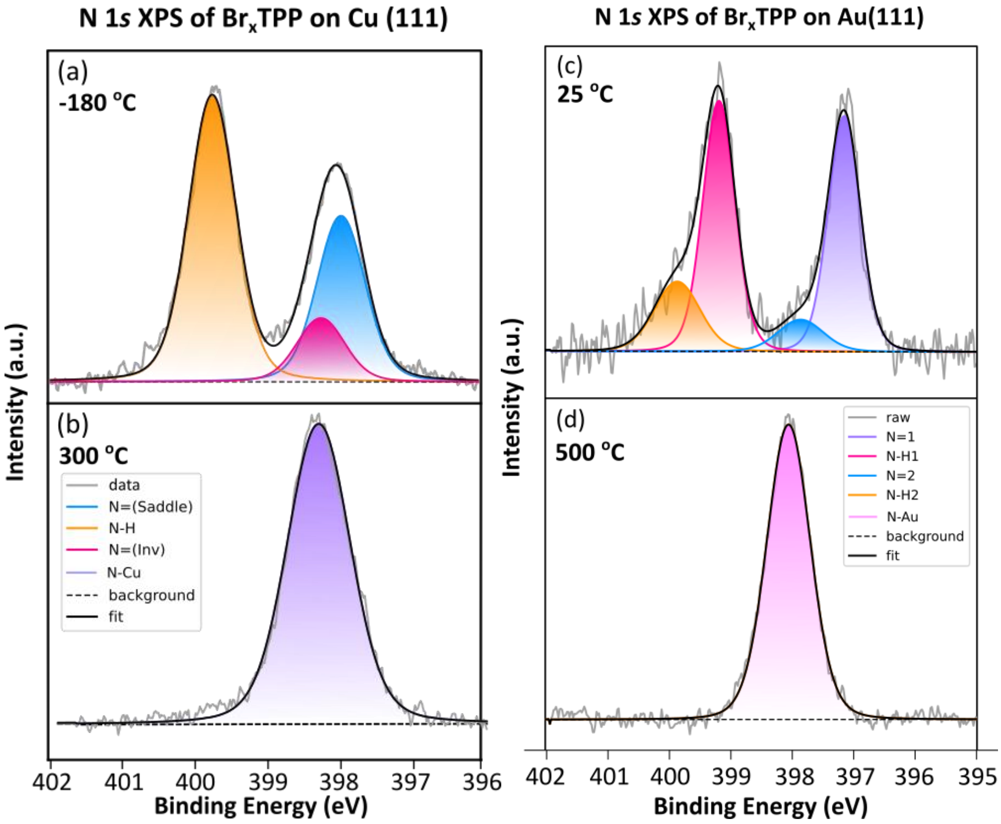

Figure S9: N 1*s* XPS region. High resolution XPS measurements and peak fitting for initial and final states of reaction. (a,b) Br_xTPP on Cu(111) N 1*s* core level, photon energy= 500 eV, (c,d) Br_xTPP on Au(111) N 1*s* core level, photon energy= 500 eV.

Table S5: BE of peaks assigned to N 1s region in Figure S8

Au(111)	Iminic Nitrogen (N=)		Aminic Nitrogen (N-H)		Iminic Nitrogen (N=) B		Aminic Nitrogen (N-H) B		Metalated Nitrogen (N-Au)	
	**BE (eV)**	**FWHM**	**BE (eV)**	**FWHM**	**BE (eV)**	**FWHM**	**BE (eV)**	**FWHM**	**BE (eV)**	**FWHM**
**25 °C**	397.14	0.62	399.20	0.62	397.81	0.86	399.9	0.86		
**500 °C**									398.04	0.93

Cu(111)	Iminic Nitrogen (N=)		Inverted Nitrogen (N-Inv)		Aminic Nitrogen (N-H)		Metalated Nitrogen (N-Cu)	
	**BE (eV)**	**FWHM (eV)**	**BE (eV)**	**FWHM (eV)**	**BE (eV)**	**FWHM (eV)**	**BE (eV)**	**FWHM (eV)**
**-180 °C**	397.91	0.80	398.22	0.79	399.74	0.90		
**300 °C**							398.25	1.04

Details of NEXAFS characterisation: Peak assignment and discussion of N K-edge spectra

Table S6: C K-edge NEXAFS peak positions and intensities.

**Cu**		**Resonance Energy (eV)**				
**C K-edge**		284.2	285.2	286.5	287.3	288.8
**T (°C)**	angle of incident x-rays	1	2	3	4	5
**-180**	30	0.89	2.23	n/a	1.17	1.18
	45	0.62	1.69	n/a	1.08	1
	60	0.4	1.25	n/a	1.02	0.84
	75	0.25	0.92	n/a	1	0.73
	90	0.17	0.83	n/a	0.95	0.67
**0**	30	n/a	1.89	1.5	n/a	1.25
	45	n/a	1.31	1.18	n/a	0.98
	60	n/a	0.81	0.96	n/a	0.74
	75	n/a	0.43	0.77	n/a	0.58
	90	n/a	0.3	0.72	n/a	0.5
**100**	30	n/a	1.28	1.17	0.95	n/a
	45	n/a	1.33	1.21	0.91	n/a
	60	n/a	1.411	1.26	0.826	n/a
	75	n/a	1.411	1.26	0.729	n/a
	90	n/a	1.31	0.89	0.546	n/a

**Au**		**Resonance Energy (eV)**					
**C K edge**		284.2	285.3	287.3	287.8	288.8	290
**T (°C)**	angle of incident x-rays	1	2	3	4	5	6
**20**	30	1.451	2.745	1.12	1.23	1.365	n/a
	45	0.989	2.18	0.862	0.907	1.054	n/a
	60	0.608	1.623	0.653	0.641	0.797	n/a
	75	0.293	1.194	0.506	0.465	0.625	n/a
	90	0.17	0.981	0.445	0.387	0.531	n/a
**290**	30	1.917	2.546	1.371	1.447	1.462	n/a
	45	1.235	1.852	0.943	0.988	1.075	n/a
	60	0.662	1.272	0.609	0.62	0.749	n/a
	75	0.268	0.833	0.366	0.378	0.526	n/a
	90	0.131	0.685	0.283	0.279	0.454	n/a

**350**	30	2.451	2.451	n/a	1.613	1.653	n/a
	45	1.529	1.657	n/a	1.088	1.22	n/a
	60	0.786	0.932	n/a	0.647	0.866	n/a
	75	0.287	0.451	n/a	0.363	0.673	n/a
	90	0.101	0.225	n/a	0.254	0.56	n/a
**500.1**	30	2.502	2.605	n/a	1.559	n/a	1.663
	45	1.502	1.587	n/a	1.059	n/a	1.201
	60	0.692	0.786	n/a	0.598	n/a	0.777
	75	0.211	0.268	n/a	0.343	n/a	0.494
	90	0.013	0.032	n/a	0.249	n/a	0.381
500.2	30	2.428	2.366	n/a	1.66	n/a	1.711
	45	1.523	1.541	n/a	1.106	n/a	1.327
	60	0.755	0.781	n/a	0.639	n/a	0.965
	75	0.249	0.277	n/a	0.328	n/a	0.748
	90	0.068	0.086	n/a	0.216	n/a	0.592

N K-edge NEXAFS

The angle resolved NEXAFS data for the N K-edge (Fig. S10) shows an evolution of the π* states at different annealing steps. On both Au(111) and Cu(111) surfaces the change in peak positions and intensities represent changes in electronic structure due to the ring-closing and metalation steps as detailed in the main manuscript. Analysis of the 'tilt-angle' relative to the surface (Table S7 – procedure as for the C K-edge data, detailed in the main manuscript) indicates little during annealing: 22-39° on Au(111), and 49-55° on Cu(111). As it is non-trivial to assign the resonance in NEXAFS to specific chemical bonds (due to the transition between the free-base Br_xTPP, the ring-closed variant, and the metalated form) specific structural changes are not assigned based upon this data.

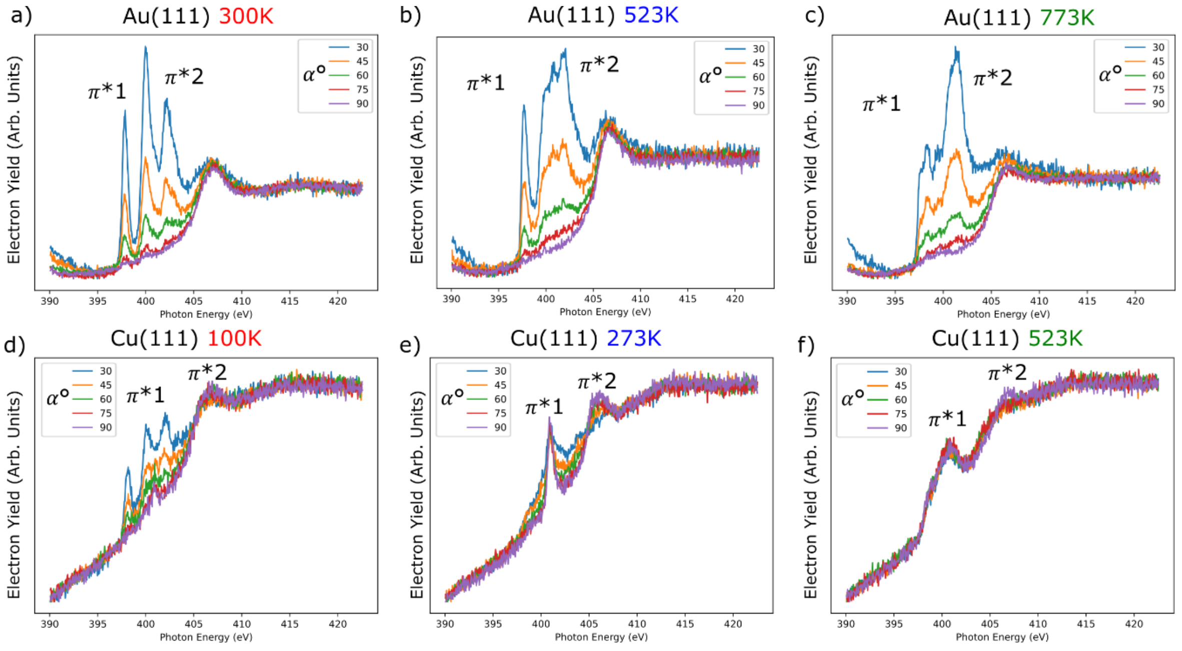

Figure S10: NEXAFS spectra at the N K-edge for Br_xTPP on Au(111) (a-c) and Cu(111) (d-f).

Table S7- Calculated angles of π* orbitals to the surface normal for different N K-edge NEXAFS resonance peaks.

Region	Surface	Temp (K)	π*1 ϴ°	π*2 ϴ°
N K-edge	Au (111)	300	21.7±0.1	24.0±0.2
N K-edge	Au (111)	523	23.4±0.2	26.7±0.5
N K-edge	Au (111)	773	29.3±0.9	25.5±0.4
N K-edge	Cu (111)	100	49.0±0.2	55.7±0.3
N K-edge	Cu (111)	273	55.9±0.2	57.1±0.2
N K-edge	Cu (111)	573	55.7±0.2	55.9±0.5

REFERENCES

(1) Grill, L.; Dyer, M.; Lafferentz, L.; Persson, M.; Peters, M. V.; Hecht, S. Nano-Architectures by Covalent Assembly of Molecular Building Blocks. *Nat. Nanotechnol.* **2007**, *2* (11), 687–691. https://doi.org/10.1038/nnano.2007.346.
(2) Tschakert, J.; Zhong, Q.; Martin-Jimenez, D.; Carracedo-Cosme, J.; Romero-Muñiz, C.; Henkel, P.; Schlöder, T.; Ahles, S.; Mollenhauer, D.; Wegner, H. A.; Pou, P.; Pérez, R.; Schirmeisen, A.; Ebeling, D. Surface-Controlled Reversal of the Selectivity of Halogen Bonds. *Nat. Commun.* **2020**, *11* (1), 5630. https://doi.org/10.1038/s41467-020-19379-4.
(3) Jarvis, S. P.; Taylor, S.; Baran, J. D.; Thompson, D.; Saywell, A.; Mangham, B.; Champness, N. R.; Larsson, J. A.; Moriarty, P. Physisorption Controls the Conformation and Density of States of an Adsorbed Porphyrin. *J. Phys. Chem. C* **2015**, *119* (50), 27982–27994. https://doi.org/10.1021/acs.jpcc.5b08350.
